# Evaluation of Salivary Cytokines for Diagnosis of both Trauma-Induced and Genetic Heterotopic Ossification

**DOI:** 10.3389/fendo.2017.00074

**Published:** 2017-04-24

**Authors:** Hsiao Hsin Sung Hsieh, Michael T. Chung, Ronald M. Allen, Kavitha Ranganathan, Joe Habbouche, David Cholok, Jonathan Butts, Arminder Kaura, Ramkumar Tiruvannamalai-Annamalai, Chris Breuler, Caitlin Priest, Shawn J. Loder, John Li, Shuli Li, Jan Stegemann, Steven L. Kunkel, Benjamin Levi

**Affiliations:** ^1^Burn/Wound and Regenerative Medicine Laboratory, Department of Surgery, University of Michigan, Ann Arbor, MI, USA; ^2^Experimental Rheumatology, Radboud University Medical Center, Nijmegen, Netherlands; ^3^Department of Pathology, University of Michigan, Ann Arbor, MI, USA; ^4^Department of Biomedical Engineering, University of Michigan, Ann Arbor, MI, USA

**Keywords:** biomarkers, inflammatory cytokines, heterotopic ossification, saliva, minimally invasive, fibrodysplasia ossificans progresiva

## Abstract

**Purpose:**

Heterotopic ossification (HO) occurs in the setting of persistent systemic inflammation. The identification of reliable biomarkers can serve as an early diagnostic tool for HO, especially given the current lack of effective treatment strategies. Although serum biomarkers have great utility, they can be inappropriate or ineffective in traumatic acute injuries and in patients with fibrodysplasia ossificans progressiva (FOP). Therefore, the goal of this study is to profile the cytokines associated with HO using a different non-invasive source of biomarkers.

**Methods:**

Serum and saliva were collected from a model of trauma-induced HO (tHO) with hind limb Achilles’ tenotomy and dorsal burn injury at indicated time points (pre-injury, 48 h, 1 week, and 3 weeks post-injury) and a genetic non-trauma HO model (*Nfatc1-Cre/caAcvr1^fl/wt^*). Samples were analyzed for 27 cytokines using the Bio-Plex assay. Histologic evaluation was performed in *Nfatc1-Cre/caAcvr1^fl/wt^* mice and at 48 h and 1 week post-injury in burn tenotomy mice. The mRNA expression levels of these cytokines at the tenotomy site were also quantified with quantitative real-time PCR. Pearson correlation coefficient was assessed between saliva and serum.

**Results:**

Levels of TNF-α and IL-1β peaked at 48 h and 1 week post-injury in the burn/tenotomy cohort, and these values were significantly higher when compared with both uninjured (*p* < 0.01, *p* < 0.03) and burn-only mice (*p* < 0.01, *p* < 0.01). Immunofluorescence staining confirmed enhanced expression of IL-1β, TNF-α, and MCP-1 at the tenotomy site 48 h after injury. Monocyte chemoattractant protein-1 (MCP-1) and VEGF was detected in saliva showing elevated levels at 1 week post-injury in our tHO model when compared with both uninjured (*p* < 0.001, *p* < 0.01) and burn-only mice (*p* < 0.005, *p* < 0.01). The Pearson correlation between serum MCP-1 and salivary MCP-1 was statistically significant (*r* = 0.9686, *p* < 0.001) Similarly, the Pearson correlation between serum VEGF and salivary VEGF was statistically significant (*r* = 0.9709, *p* < 0.05).

**Conclusion:**

In this preliminary study, we characterized the diagnostic potential of specific salivary cytokines that may serve as biomarkers for an early-stage diagnosis of HO. This study identified two candidate biomarkers for further study and suggests a novel method for diagnosis in the context of current difficult diagnosis and risks of current diagnostic methods in certain patients.

## Introduction

Accurate diagnoses of progressively debilitating disease and sequelae of severe traumatic injury are imperative for early treatment and improved patient outcomes. Unfortunately, misdiagnoses and delayed diagnoses are made at an alarming rate with severe consequences, particularly with presentation of rare disease. The formation of heterotopic ossification (HO), endochondral bone within soft tissue structures (1), provides a unique pathology representative of this dilemma. Clinically, HO can result from multiple etiologies including severe burns, neurologic injury, major surgery, and genetic mutation (2). In the case of fibrodysplasia ossificans progressiva (FOP), an autosomal dominant disease in which patients develop accumulating foci of HO in response to local and systemic insult, 87% of patients were found to be incorrectly diagnosed at initial presentation (3). Of these, 49% of patients reported accelerated bone development from invasive and unnecessary diagnostic procedures. These patients will develop HO with even as minor an insult as a blood draw highlighting the need for accurate and non-invasive diagnostic tools (3). Even with a diagnosis of FOP, clinicians are still unable to definitively predict when a new HO lesion will develop.

Trauma-induced HO (tHO) patients represent an equally challenging diagnostic dilemma as they do not have a genetic mutation as seen in FOP. HO occurs in over 20% of primary hip replacements, extremity traumas, amputations, large total body surface area burns, traumatic brain injuries, spinal cord injuries, and pressure ulcers and over 65% of repeat hip replacements and blast injuries (4–6). Contemporary treatment protocols involve surgical extirpation; however, even after a technically successful operation, over 75% of patients have restricted range of motion and 30% of patients have recurrence (7, 8). Early detection and timed treatments are needed for patients at high risk for HO, given the sub-optimal outcomes under the standard treatment paradigm. Thus, alternative strategies are needed to predict which patients will develop HO. Therefore, the primary translational gap to prevent this complication of trauma is early diagnosis and access to a prophylactic agent that can be safely administered to appropriate candidates.

Anatomic HO sites elude radiographic detection prior to 3 weeks post-injury by which point occupational therapy must be halted and joint contractures progress. Clinical detection is performed with computed tomography (takes an average of 23 days after symptom development to show evidence of HO) (9); bone scans with 99mTc-MDP (low specificity, which leads to potential difficulties in discriminating HO from other inflammatory, traumatic, or degenerative processes of the skeleton) (10); ultrasound (relies on the expertise of the physician, the availability of ultrasound equipment) (11); and serum ALP measurement (sensitive, but not specific, with alterations dependent on hepatic and renal function) (12).

Recent studies in HO have detected matrix metalloproteinase-9 as an early-stage biomarker in a mouse model of BMP-induced HO (13). Other studies in human serum and wound effluent from patients following traumatic injury have demonstrated the prognostic correlation of HO development with elevated concentrations of multiple cytokines and biomarkers including interleukin-6 (IL-6), interleukin-10 (IL-10), monocyte chemoattractant protein-1 (MCP-1), interferon γ-induced protein 10, and macrophage inflammatory protein-1α (MIP-1α) (14, 15). These findings support the utility of using biomarkers in HO diagnosis utilizing sample collection from serum and wound effluent (15).

Serum biomarkers have great utility due to the fact that they are easily accessible and minimally invasive to patients. However, serum biomarkers have been described for wound healing in chronically malnourished elderly or chronically debilitated patients with modest success and have been found inappropriate or ineffective in traumatic acute injuries, as seen with combat casualties (16). For such patients with complex trauma, appropriate biomarkers of local (*via* wound effluent) wound healing are on the horizon (17). Blood collection is invasive, requires pre-processing, and entails higher risk of contracting infectious disease. Alternatively, saliva is a mirror of the body’s health as a wide spectrum of biomolecules is transported from the blood capillaries through the epithelium of salivary glands (18, 19). Saliva collection is non-invasive and less resource intensive. Also advantageous, saliva possesses lower protein content, which would be potentially confounding, and less variation in terms of composition when compared to serum (20). Saliva biomarkers have been used for assays of bone turnover biomarkers (21) and also have been increasingly used in the diagnosis, prevention, and treatment of multiple diseases including certain cancers, cardiovascular disease, diabetes, and graft-versus-host disease (GVHD) (22). In particular, the cytokine interleukin-1β (IL-1β) was shown to be elevated in patients with oral cancer, while low to undetectable in serum at the same time points (23). Similarly, in patients with GVHD, IL-1β was detectable in saliva before the time of diagnosis and remained elevated for a significantly longer duration than serum levels (24). Here, we demonstrate the diagnostic utility of saliva biomarkers including MCP-1 and IL-1β for HO development using tHO and non-trauma genetic HO mouse models with clinical translatability to burn trauma patients who go on to develop HO.

## Materials and Methods

### Animals

All animals were housed in standard conditions. Animal care was provided in accordance with the University of Michigan School of Medicine guidelines and policies for the use of laboratory animals. C57BL/6 male mice aged 6–8 weeks (Charles River, Wilmington, MA, USA) were used for all experiments describing burn or burn tenotomy. For our genetic model, mice carrying the floxed constitutively active allele of ACVR1 (*ACVR1* carrying the Q207D mutation, *ca-ACVR1^fl/wt^*) and *Nfatc1-Cre* transgenic mice were used for breeding as previously described (25–28). Resulting pups carrying both transgenes (*Nfatc1-Cre/caAcvr1^fl/wt^*) were used as experimental mice. Littermates missing one or both transgenes were used as controls.

### Trauma-Induced HO

Mice in burn-only and burn/tenotomy groups received a 30% total body surface area partial thickness burn injury to their dorsum. Briefly, mice were anesthetized with 3–5% inhaled isoflurane, and the left dorsum hair was clipped. Preoperative analgesia was provided with subcutaneous buprenorphine. The shaved area was then exposed to a metal block for 18 s that was heated to 60°C in a hot-water bath. Mice in the burn/tenotomy group then received a sharp dissection of the left Achilles tendon with sterile scissors immediately distal to the fuse point of the fibula and tibia. The tenotomy site was closed with a single 5-0 vicryl stitch placed through the skin only. After injury, all mice were allowed to recover and return to normal activity. Pain management was achieved with subcutaneous injections of buprenorphine every 12 h for 3 days.

### Sample Collection

Saliva, serum, and tendon tissue were collected (Figure S1 in Supplementary Material) at four time points (pre-injury, 48 h, 1 week, and 3 weeks post-burn tenotomy) from a model of tHO with hind limb Achilles’ tenotomy and dorsal burn injury. Saliva and serum were also collected from a genetic non-trauma HO model (*Nfatc1-Cre/caAcvr1^fl/wt^*). Anesthesia was induced by intramuscular injection of 60 mg/ml ketamine and 8 mg/ml xylazine at a dose of 1 µl/kg body weight. Salivation was induced by subcutaneous injection of pilocarpine (0.05 mg pilocarpine/100 g body weight). Saliva was obtained from the oral cavity and immediately placed in pre-chilled 1.5-ml microcentrifuge tubes. The samples were stored in −80°C until analysis. After the mice were sacrificed, blood was collected in a Serum Gel Z/1.1 tube, and serum was extracted by centrifugation at 14,000 × *g* for 10 min. Tendon tissue from the tenotomy site was removed from mice, snap-frozen in liquid, and stored in −80°C until analysis.

### Histological Examination

Tissue from the tenotomy site was fixed with 10% buffered formalin overnight at 4°C followed by paraffin embedding. The 5-µm sections were cut and mounted on Superfrost Plus Slides (Fisher Scientific, Hampton, NH, USA) and stored at room temperature. Immunostaining was performed on rehydrated wax sections with the following antibodies: anti-mouse TNF-α (sc-1350, Santa Cruz Biotechnology, Dallas, TX, USA), anti-mouse IL-1β (sc-7884, Santa Cruz Biotechnology), and anti-mouse MCP-1 (ab25124, Abcam, Cambridge, UK). Appropriate dilutions were determined before achieving final images. The appropriate fluorescent secondary antibody was applied and visualized using fluorescence microscopy. Secondary antibodies consisted of Alexa Fluor488 anti-rabbit (A21206, Life Technologies, Carlsbad, CA, USA) and Alexa Fluor488 anti-goat (A11055, Life Technologies).

### Microscopy

All fluorescently stained images were taken using an Olympus BX-51 upright light microscope equipped with standard DAPI, 488 nm, and TRITC cubes attached to an Olympus DP-70 high-resolution digital camera. Each site was imaged in all channels and overlaid in DPViewer before examination in Adobe Photoshop (Adobe Systems, San Jose, CA, USA).

### RNA Extraction and Quantitative Real-time PCR (qPCR)

Tissue samples were submerged in TRIzol (Invitrogen, Carlsbad, CA, USA; 1 ml of TRIzol/50–100 mg of tissue). Tissues were homogenized in the TRIzol solution until no visible particles remained. Addition of chloroform (Sigma-Aldrich, St. Louis, MO, USA) and subsequent centrifugation at 14,000 × *g* separated the mixture into three phases (lower red phenol/chloroform phase, interphase, and upper aqueous phase). RNA was then extracted from the upper aqueous phase using the RNAeasy kit (Qiagen, Hilden, Germany) centrifuged at 7,500 × *g*, air dried, resuspended in RNase-free water, and analyzed. Reverse transcription was performed with 1 µg RNA using High Capacity cDNA Reverse Transcription kit (Applied Biosystems, Foster City, CA, USA). qPCR for four genes, *Mcp-1, Tnf-α, Il-1β*, and *Il-6*, normalized with the housekeeping gene (*Tbp*) was then carried out in triplicate in reaction volumes of 20 µl using SYBR Green Master Mix (Applied Biosystems, Foster City, CA, USA) for 15 min at 95°C for initial denaturing, followed by 40 cycles of 95°C for 30 s and 60°C for 30 s in the ABI 7500HT Fast Real-Time PCR system. Specific primers for these genes were chosen based on their PrimerBank sequence (Figure S2 in Supplementary Material).

### Multiplex Cytokine Analysis

Concentrations of 27 (IL-1β, IL-2, IL-3, IL-4, IL-5, IL-6, IL-8, IL-9, IL-10, IL-12, IL-13, eotaxin, G-CSF, CxCl-1, TNF-α, MCP-1, M-CSF, MIG, MIP-1α, MIP-1β, MIP-2, KC, IFN-γ, G-CSF, GM-CSF, RANTES, and VEGF) cytokines in serum and saliva were analyzed using a Luminex Bio-Plex 200 system (Bio-Rad, Hercules, CA, USA) according to the manufacturer’s protocol, as previously described (29). For cytokine analysis, mouse stock cytokines of known concentrations (provided with the kit) were used to generate standard curves. The threshold of each cytokine was routinely <5 pg/ml.

### Statistical Analysis

Means and SDs were calculated from numerical data, as presented in the text, figures, and figure legends. In figures, bar graphs represent means and error bars represent 1 SD. Statistical analysis was performed using Student’s *t*-test to directly compare two groups. Inequality of SDs was excluded by using the Levene’s test. Pearson correlation coefficient test was used to compare the correlation between saliva and serum. *p* Values <0.05 were regarded as statistically significant.

## Results

### Serum Cytokines in Mouse Burn Tenotomy Model

**Figure 1:**
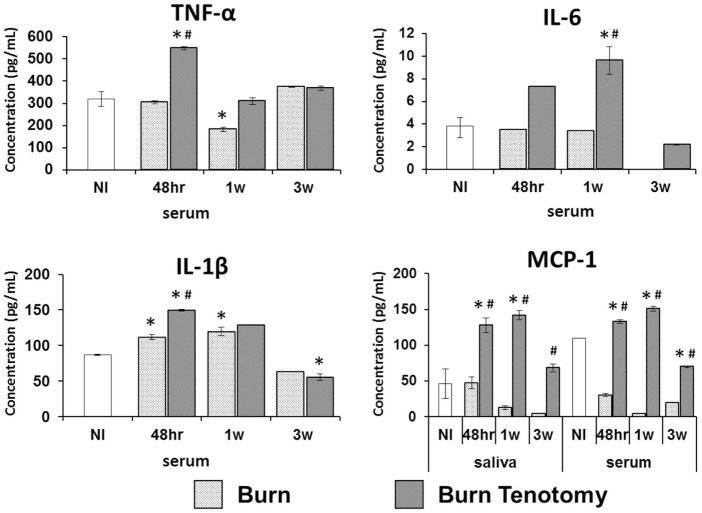
**Multiplex analysis of serum and saliva in injury model of heterotopic ossification**. Graphs depict concentrations of monocyte chemoattractant protein-1 (MCP-1) in saliva and serum, and concentrations of interleukin-6 (IL-6), interleukin-1β (IL-1β), and TNF-α in serum from mice 48 h, 1 week, and 3 weeks post-injury (burn or burn tenotomy) compared to non-injured (NI) mice (*p < 0.05 or compared to burn mice ^#^*p* < 0.05).

Inflammatory cytokines are reliably upregulated in patients preceding formation of heterotopic bone. In our burn tenotomy model, HO formation occurs exclusively with concomitant transection and not with burn alone, despite systemic inflammation in both groups. In order to assess departures in cytokine profiles specific to bone formation, serum was collected in burn and burn/tenotomy mice at four time points: pre-injury, 48 h, 1 week, and 3 weeks post-injury. Cytokines and growth factors were assessed using the multiplex bead assay. Levels of TNF-α and IL-1β peaked at 48 h in the burn/tenotomy cohort, and these values were significantly higher when compared with both uninjured (*p* < 0.01, *p* < 0.03) and burn-only mice (*p* < 0.01, *p* < 0.01). Similarly, IL-6 increased at 1 week post-injury in the burn/tenotomy cohort, and this value was significantly higher when compared with both uninjured (*p* < 0.01) and burn-only mice (*p* < 0.04). MCP-1 levels in the serum were increased at 48 h and 1 week post-injury in the burn/tenotomy group, and these values were significantly higher when compared with both uninjured (*p* < 0.007, *p* < 0.02) and burn-only mice (*p* < 0.001, *p* < 0.005) (Figure 1).

### Salivary Cytokines in Burn Tenotomy Model

Blood serum from venipuncture has been broadly used to determine cytokine levels in the bloodstream. However, serum extraction is an invasive procedure. Thus, we sought to determine the presence of these serum cytokines in saliva, a non-invasive diagnostic tool. MCP-1 was detected in saliva showing elevated levels at 1 week post-injury in our tHO model when compared with both uninjured (*p* < 0.001, *p* < 0.01) and burn-only mice (*p* < 0.005, *p* < 0.01) (Figure 1). However, saliva levels of MCP-1 in burn-only mice were not significantly greater than corresponding levels in uninjured mice. The Pearson correlation between serum MCP-1 and salivary MCP-1 was statistically significant (*r* = 0.9686, *p* < 0.001) similarly. However, TNF-α, IL-1β, and IL-6 levels were very low in saliva and barely detectable with the multiplex bead assay in both burn-only mice and burn/tenotomy mice (data not shown).

### Tissue Expression of Cytokines

**Figure 2:**
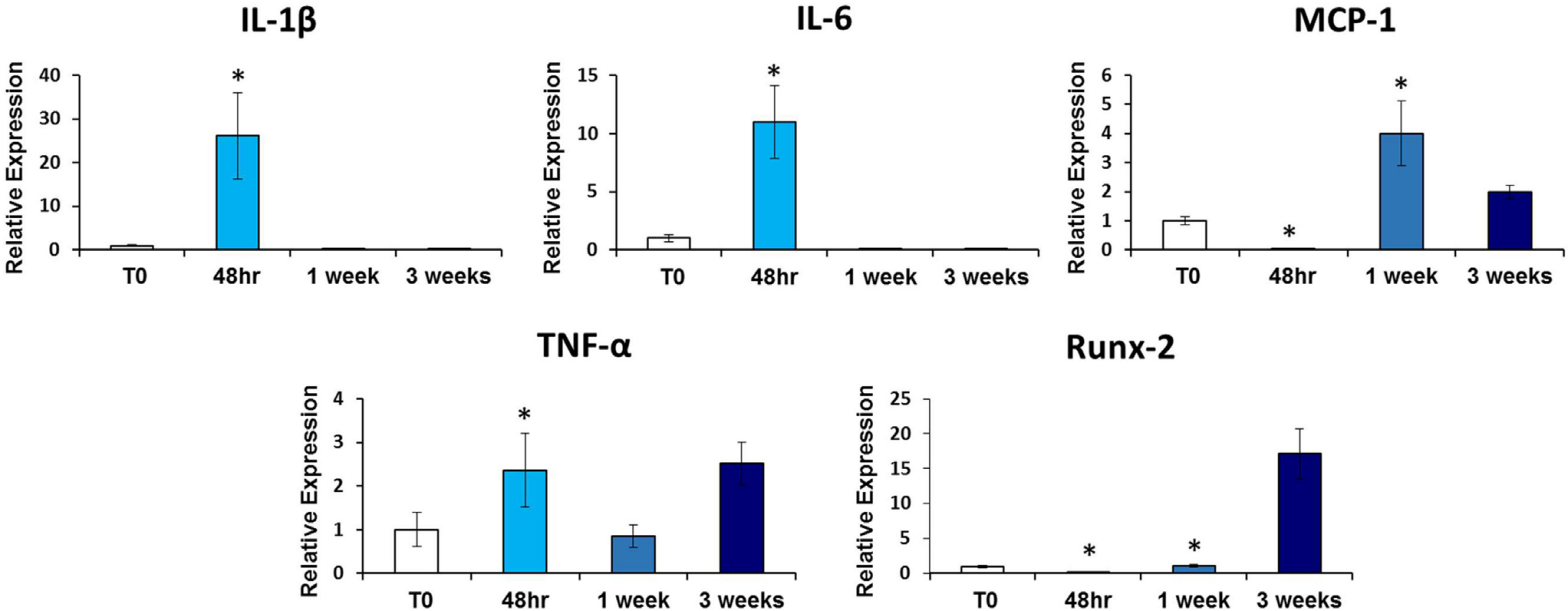
**qRT-PCR analysis of heterotopic ossification (HO) site tendon tissue**. Graphs depict relative expression of indicated genes in tendon tissue from the HO site 48 h, 1 week, and 3 weeks post-injury compared to tendon tissue from non-injured (NI) mice. Data normalized to *Gapdh* expression (*p < 0.05).

Given that inflammatory cytokines are involved in tHO formation, we investigated the mRNA expression levels of these cytokines at the tenotomy site at the same time point to see if there was a correlation with salivary cytokine levels. We found increased expression levels of *Tnf-*α (*p* < 0.02), *Il-1*β (*p* < 0.01), *Il-6* (*p* < 0.003), and *Mcp-1* (*p* < 0.01) at 48 h post-injury in burn/tenotomy mice compared to the uninjured cohort, concordant with levels of these cytokines in serum and/or saliva (Figure 2).

### Salivary and Serum Cytokines in Genetic Non-Traumatic Model of HO

**Figure 3:**
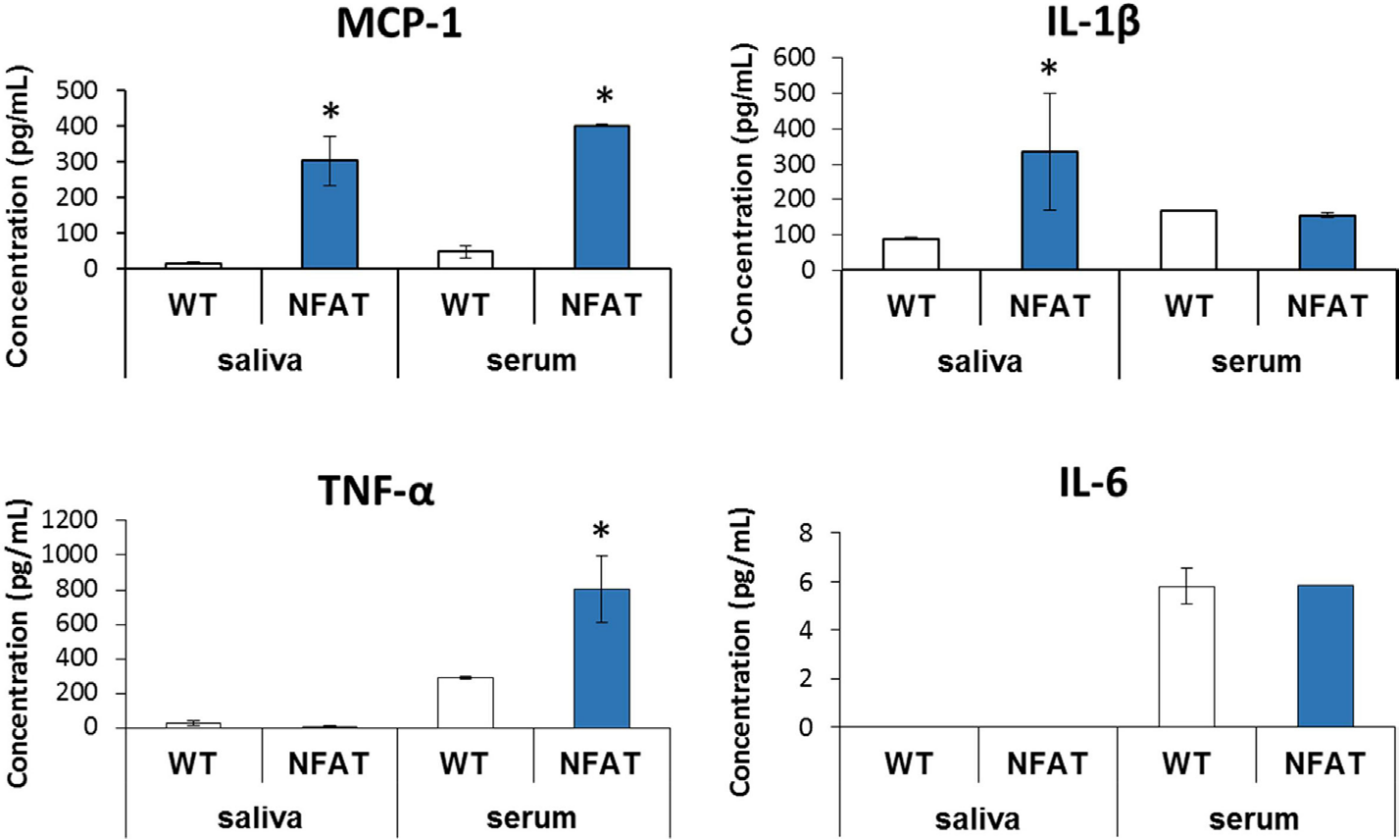
**Multiplex analysis of serum and saliva in genetic model of heterotopic ossification**. Graphs depict concentration of indicated proteins in saliva and serum [monocyte chemoattractant protein-1 (MCP-1) and interleukin-1β (IL-1β)] or in serum alone [TNF-α, interleukin-6 (IL-6)] in wild-type mice vs. genetic non-trauma model (NFAT) (*p < 0.05).

*Nfatc1-Cre/caAcvr1^fl/wt^* mice have been shown to form HO spontaneously similar to that seen in genetic forms of HO or FOP (30). These mice allow greater assessment of cytokines without the dramatic effect of local trauma. Mutant mice demonstrated increased levels of MCP-1 (*p* < 0.01) in the serum and saliva. However, IL-1β was increased only in saliva (*p* < 0.002) (Figure 3). Conversely, TNF-α was detectable only in serum, with values significantly greater in the mutant than in control mice (*p* < 0.03). IL-6 showed no differences in serum between the mutant and the littermate controls and undetectable in the saliva for both groups.

### Immunostaining for Inflammatory Cytokines in HO

**Figure 4:**
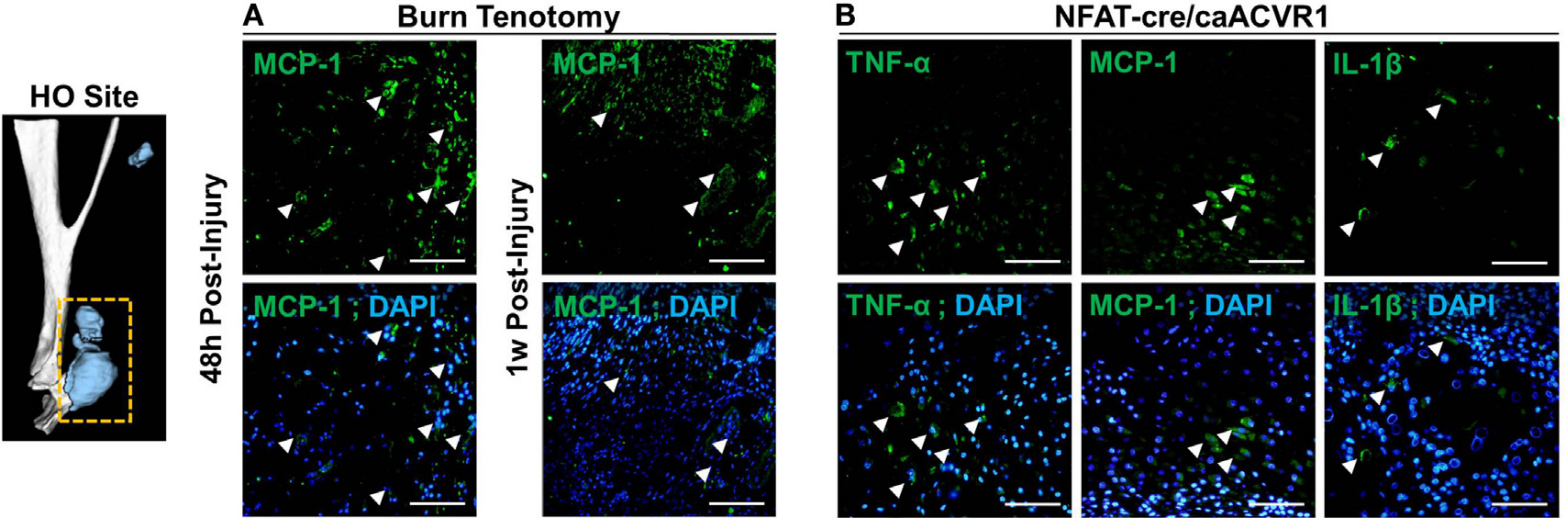
**Immunostaining of cytokines in the heterotopic ossification (HO) site**. **(A)** Images show immunofluorescence staining of indicated cytokines (green) at the site of injury at 48 h and 1 week post-burn/tenotomy. **(B)** Images show immunofluorescence staining of indicated cytokines (green) HO anlagen of the genetic model NFAT-cre caACVR with corresponding DAPI overlays. Arrows indicate examples of positive staining. Scale bars indicate 50 µm.

We next used immunostaining to assess localization of cytokine alterations. Immunofluorescence staining confirmed enhanced expression of MCP-1 (Figure 4) at the injury site 48 h after injury in burn/tenotomy mice compared to 1 week. This corresponds to the qPCR data showing increased expression of MCP-1 at the injury site specifically at 48 h, but not at 1 week. Furthermore, TNF-α, IL-1β, and MCP-1 were localized to the areas of cartilage formation within the HO anlagen of *Nfatc1-Cre/caAcvr1^fl/wt^* mice, a transgenic model in which ectopic bone forms progressively in both soft tissue and joint spaces. This supports the hypothesis that HO production in genetic HO-forming mutations is incited by microtrauma, which leads to inflammatory signaling.

## Discussion

Heterotopic ossification leads to bone deposition in extraskeletal sites severely restricting range of motion and causing chronic pain and wounds. HO occurs in severe combat-injured/blast injury patients and in over 65% of repeat hip replacement patients and (4, 6, 31). Advances in critical care medicine have improved the survival of poly-trauma and large total body surface area burn patients, causing a concomitant rise in the number of patients at risk for HO. Therefore, there is a critical need to detect HO before it occurs, especially given the current lack of effective treatment. Assessing biomarkers in an overall diagnostic strategy may allow earlier diagnosis and HO prevention.

Molecular biomarkers have proven valuable in diagnosing and monitoring disease progression and response to therapies in various disease states. The clinical course of trauma patients is heavily influenced by the systemic inflammatory response, in which the main pro-inflammatory cytokines IL-1β, IL-6, and TNF-α are involved (32, 33). Activation of inflammatory pathways through the innate immune system also appears to be an important trigger for flare-ups of FOP (34, 35). Serum analysis in our tHO model and genetic non-trauma model (*Nfatc1-Cre/caAcvr1^fl/wt^*) demonstrated a pronounced inflammatory state with increased concentrations of TNF-α and IL-1β at 48 h and 1 week post-burn tenotomy compared to uninjured and burn-only mice. This was confirmed with mRNA levels in our study and is consistent with previous studies that have identified TNF-α and IL-1β as crucial inflammatory cytokines in bone healing and MSC osteogenic differentiation (36–38). However, levels of IL-6 were not associated with the development of HO, as previously observed in human (14). Nonetheless, the results support the hypothesis that HO is associated with a hyper-inflammatory systemic and local response.

Recent studies have shown that saliva actually contains a variety of molecular analytes and that these salivary constituents may actually be effective indicators of both local and systemic disorders (39–42). Although elevated serum levels of TNF-α and IL-1β were associated with the development of HO, salivary levels of these cytokines did not demonstrate a significant correlation with ectopic bone formation. However, levels of MCP-1 were elevated in saliva at 1 week post-burn tenotomy compared to uninjured and burn-only mice. Interestingly, mRNA expression of MCP-1 at the injury site was elevated at 48 h post-injury, but not at 1 week. MCP-1, an inducible pro-inflammatory chemokine, stimulates the chemotaxis of monocytes and other inflammatory cells to sites of bone injury and remodeling (43). Once activated, mononuclear phagocytes enhance the proliferation of osteoblasts and express pro-osteogenic stimuli to neighboring cells (43, 44) The damage to mesenchymal tissue at the site of injury, including periosteum, tendon, and fat, may lead to elevated serum and saliva levels of MCP-1 (45). MCP-1 signaling may then recruit circulating inflammatory cells from the bone marrow and other reservoirs, and this interaction could be a major foundation for immunological involvement in HO formation.

Fibrodysplasia ossificans progressiva is a rare and disabling genetic condition characterized by progressive HO in specific anatomic patterns. Nearly 90% of FOP patients worldwide are initially misdiagnosed and 67% undergo dangerous and unnecessary diagnostic procedures that lead to permanent harm and lifelong disability as a consequence (46). Minor trauma, such as blood draw, can trigger painful new flare-ups of FOP leading to progressive HO. Therefore, in this subset of patients, a different source for biomarkers would be more appropriate. This study advances the concept of using a different source of biomarkers, such as saliva, in this subset of patients who require alternatives to the current standards of care. Furthermore, we have identified a number of biomarkers that could potentially be used to improve upon the current challenge of effectively diagnosing HO during a window in which it can be intervened upon.

The main limitation of this study is the relatively small sample size. However, this study showed unique local and systemic inflammatory profiles associated with formation of HO in a tHO model and genetic non-trauma model. Second, a majority of patients who develop HO typically sustain multiple injuries, which may influence the amount of detectable cytokines by generating and maintaining systemic inflammatory responses. Thus, the findings presented in this study may not be applicable to all patients who develop HO. In summary, a systemic inflammatory state, as evident by elevated levels of inflammatory cytokines and chemokines, is associated with the development of HO. This study identified two candidate biomarkers for further study and suggests a novel method for diagnosis in the context of current difficult diagnosis and risks of current diagnostic methods in certain patients. However, future efforts geared toward modeling these data must account for their complex, time-dependent, and non-linear nature.

## Ethics Statement

Procedures involving animals were approved by the Institutional Animal Care and Use Committee of the University of Michigan (PRO0005909).

## Author Contributions

Conception or design of the work: HH, MC, and BL. Data collection: HH, RTA, JB, AK, RMA, CB, CP, and JL. Data analysis and interpretation: HH, MC, JH, DC, KR, RTA, SJL, BL, and SL. Drafting the article: HH, MC, KR, DC, and JH. Critical revision of the article: JS, SK, BL, and SJL. Final approval of the version to be published: JS, SK, and BL.

## Conflict of Interest Statement

The authors declare that the research was conducted in the absence of any commercial or financial relationships that could be construed as a potential conflict of interest. BL collaborates with Boehringer Ingelheim on a project not examined in this study. None of the authors have a financial interest in any of the products, devices, or drugs mentioned in this manuscript.

## Supplementary Material

Figure S1 **Saliva, serum, and tendon tissue were collected from mice which received a 30% total body surface area partial-thickness dorsal burn injury with hindlimb Achilles’ tendon transection (trauma model) and from *Nfatc1-Cre/caAcvr1^fl/wt^* mice (genetic model)**. These samples were analyzed for cytokines of interest.

Figure S2 **Gene primers for quantitative RT-PCR**.
