# Supplementary figures and images for: Evaluation of Salivary Cytokines for Diagnosis of both Trauma-Induced and Genetic Heterotopic Ossification
Source: Front Endocrinol (Lausanne). 2017 Apr 24;8:74. doi: 10.3389/fendo.2017.00074 (PMC5401868; doi:10.3389/fendo.2017.00074)

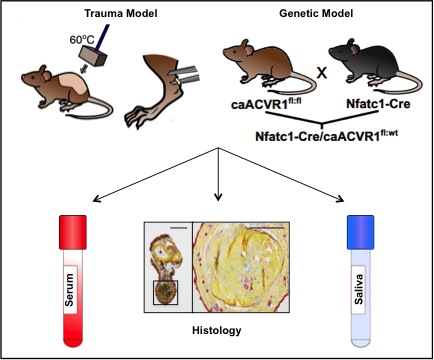

Supplement: Figure S1 — Saliva, serum, and tendon tissue were collected from mice which received a 30% total body surface area partial-thickness dorsal burn injury with hindlimb Achilles’ tendon transection (trauma model) and from Nfatc1-Cre/caAcvr1fl/wt mice (genetic model). These samples were analyzed for cytokines of interest. [file Image_1.JPEG]

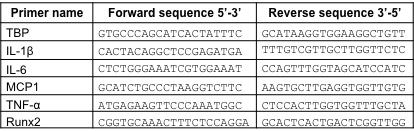

Supplement: Figure S2 — Gene primers for quantitative RT-PCR. [file Image_2.JPEG]
